# Snus and Cardiometabolic Health Markers Among Swedish Young Adults

**DOI:** 10.1093/ntr/ntae267

**Published:** 2024-11-15

**Authors:** Anna Zettergren, Niklas Andersson, Göran Pershagen, Christian Lindh, Antonios Georgelis, Inger Kull, Erik Melén, Sandra Ekström, Petter Ljungman, Anna Bergström

**Affiliations:** Institute of Environmental Medicine, Karolinska Institutet, Stockholm, Sweden; Institute of Environmental Medicine, Karolinska Institutet, Stockholm, Sweden; Institute of Environmental Medicine, Karolinska Institutet, Stockholm, Sweden; Division of Occupational and Environmental Medicine, Lund University, Lund, Sweden; Institute of Environmental Medicine, Karolinska Institutet, Stockholm, Sweden; Centre for Occupational and Environmental Medicine, Stockholm Region, Sweden; Department of Clinical Science and Education, Södersjukhuset, Karolinska Institutet, Stockholm, Sweden; Sachs’ Children and Youth Hospital, Södersjukhuset, Stockholm, Sweden; Department of Clinical Science and Education, Södersjukhuset, Karolinska Institutet, Stockholm, Sweden; Sachs’ Children and Youth Hospital, Södersjukhuset, Stockholm, Sweden; Institute of Environmental Medicine, Karolinska Institutet, Stockholm, Sweden; Centre for Occupational and Environmental Medicine, Stockholm Region, Sweden; Department of Clinical Science and Education, Södersjukhuset, Karolinska Institutet, Stockholm, Sweden; Institute of Environmental Medicine, Karolinska Institutet, Stockholm, Sweden; Department of Cardiology, Danderyd Hospital, Stockholm Sweden; Department of Global Public Health, Karolinska Institutet, Stockholm Sweden; Institute of Environmental Medicine, Karolinska Institutet, Stockholm, Sweden

## Abstract

**Introduction:**

Snus is suggested as a risk factor for cardiometabolic disease, but little is known about health effects in young populations, particularly in women. We aimed to investigate associations between snus and cardiometabolic health markers among young men and women.

**Aims and Methods:**

This study was conducted within the BAMSE (Swedish acronym for Children, Allergy, Environment, Stockholm, Epidemiology) birth cohort and included participants followed up around 24 years (*n* = 2256) and 26 years (*n* = 1011). Snus use was assessed at 24 years by questionnaires. Cardiometabolic health markers were recorded at clinical examinations at 24 and 26 years. Associations between snus use and cardiometabolic markers were assessed by multivariable linear regression.

**Results:**

Snus was used by 6.4% (*n* = 81) among women and 21.9% (*n* = 219) among men. Compared to no tobacco use, daily exclusive snus use among women at 24 years was associated with higher body mass index (BMI) (adjusted β: 1.93 kg/m^2^, 95% confidence interval [CI] = 0.54, 3.33) and waist circumference (WC) (aβ: 3.80 cm, 95% CI = 0.41, 7.18) at 24 years, and with higher BMI (aβ: 2.73 kg/m^2^, 95% CI = 0.53 to 4.93) at 26 years. Among men, using ≥4 cans/week was associated with increased BMI (aβ: 2.48 kg/m^2^, 95% CI = 0.73, 4.24) and a tendency toward increased body fat (aβ: 2.31%, 95% CI = −0.53, 5.14) at 26 years. Snus use was not associated with glycemic status or blood pressure.

**Conclusions:**

Our results suggest that snus is associated with increased BMI, and possibly other measures of adiposity, among young women and heavy-using young men. Given the cross-sectional study design, the results should be interpreted with caution.

**Implications:**

We found cross-sectional associations between snus use and measures of increased adiposity in a cohort of Swedish young adults, including BMI and WC among women and BMI among heavy snus-using men. We did not find associations between snus use and body fat %, glycemic status, or blood pressure. This is one of few studies to investigate the health effect of snus among both women and men as well as cardiometabolic health markers in young adults. Given the recent trends of increased snus use among young adults, our findings highlight the need for further research on snus on cardiometabolic health.

## Introduction

Tobacco is a major risk factor of cardiovascular disease (CVD) and CVD-related deaths.^1^ Most of this risk is attributable to cigarette smoking, while other forms of tobacco appear to have a smaller impact on CVD.^2^ However, there is growing evidence on cardiovascular and metabolic health effects of snus, a Swedish type of smokeless tobacco (Swedish snuff).^3,4^ Snus has been linked to an increased risk of type 2 diabetes,^5–8^ metabolic syndrome,^9^ ischemic stroke,^10,11^ fatal myocardial infarction,^12^ heart failure,^13^ as well as overall and CVD mortality.^14,15^ Moreover, studies indicate that snus use is related to risk factors of CVD and metabolic disease, including increased body mass index (BMI) and obesity,^16–19^ blood pressure,^18,20–22^ arterial stiffness,^23^ and waist circumference (WC).^22^ However, these findings are contradicted by studies where no associations have been found between snus and risk of CVD, including peripheral artery disease,^24^ stroke,^25^ myocardial infarction,^11,25^ heart failure, and CVD mortality^11^ Similarly, null associations have been reported between snus use and risk of type 2 diabetes,^26^ as well as metabolic syndrome and its separate components, central obesity, impaired blood lipid balance, and insulin resistance.^27^

In Sweden, rates of smoking have decreased over the past decades to a current 6% daily smoking in the adult population.^28^ Instead, prevalent use of snus has remained steady and even increased in certain age groups over this period. Among men, 20% of the adult population use snus daily, and 7% among women.^28^ In recent years, young adults have had a more dramatic increase in snus use than other age groups. This is most apparent among women in the age group 16–29 years, where daily snus use has increased from 3% in 2018 to 12% in 2022.

To date, cardiometabolic health effects of snus have predominantly been studied in middle-aged populations and largely in study samples only consisting of men. Given the need for further investigation on cardiometabolic effects of snus and the lack of studies on young adults and women, we aimed to study the association between snus use and markers of cardiometabolic health among young men and women, using data from a Swedish population-based birth cohort. Our primary aim was to assess associations with snus independent of other tobacco use.

## Methods

### Study Population

The study was conducted within the BAMSE (Swedish acronym for Children, Allergy, Environment, Stockholm, Epidemiology) cohort, a Swedish population-based birth cohort. Briefly, 4089 newborn children were recruited in the Stockholm area, Sweden, between 1994 and 1996. Follow-ups have been conducted around ages 1, 2, 4, 8, 12, 16, 24, and 26 years with questionnaires on health and lifestyle as well as clinical examinations (at 4, 8, 16, 24, and 26 years). A detailed description of the BAMSE cohort is found elsewhere.^29–31^ The current study mainly utilized data from a follow-up period 2016–2019, henceforth referred to as the 24-year follow-up, as well as a later follow-up period 2020–2021 in relation to the COVID-19 pandemic, henceforth referred to as the 26-year follow-up.^32^ From the 24-year follow-up, participants who attended the clinical examination, provided full questionnaire data on tobacco use, and who were not pregnant were included in the study population (*n* = 2256, 55% of the original cohort). Attendees of the 24-year follow-up clinical examination were invited to the 26-year follow-up.^32^ Of these, attendees of the 26-year clinical examination who provided full questionnaire data on tobacco use and who were not pregnant were included for further analysis (*n* = 1011, 25% of the original cohort). A flow chart describing the sample selection of the study populations is provided in Figure S1. The study was approved by the Swedish ethical review authority and was conducted according to the Helsinki Declaration. All participants provided written informed consent.

### Exposure Assessment

Snus use was evaluated through self-assessed questionnaires at the 24-year follow-up. Participants were asked “Do you use snus?” (Answer options: No/ No, but I used to use snus/ Yes, sometimes/ Yes, every day). Similar questions were used to assess conventional cigarette, e-cigarette, and waterpipe use, although no option for former use was available for the question on e-cigarettes and waterpipe. Daily snus users were asked how many cans of snus they used per week, and occasional snus users how many cans they used per month. Additionally, participants were asked their age in years at regular snus use debut (regularly defined as weekly use).

### Cotinine Analysis

For a subset of the 24-year follow-up population, urine samples collected at the clinical examination were analyzed for the nicotine metabolite cotinine, in order to investigate the association between tobacco use and cardiometabolic markers with an objective biomarker of exposure. A detailed description of the sample selection and analysis is provided elsewhere.^33^ Briefly, a sample of 500 women and 500 men, with an oversampling of tobacco users (51% in sample selection vs. 32% in the study population) were selected for urine analysis. Urine samples were analyzed by liquid chromatography–tandem mass spectrometry (LC-MS/MS; QTRAP 5500, AB Sciex, Framingham, MA) with a limit of detection set to 1 ng/mL. Cotinine levels were adjusted for specific gravity, measured by a digital refractometer, to adjust for urine sample dilution. After applying the inclusion criteria for the current study, 994 participants (496 women and 498 men) with cotinine data were included in the study.

### Outcome Assessment

Cardiometabolic health markers were recorded during the 24-year follow-up clinical examination by trained nurses. Weight and height were measured to the nearest 0.1 kg and 0.1 cm, respectively, and BMI was subsequently calculated as kg/m^2^. WC was measured to the nearest 1.0 cm. Body fat % was measured by bioelectric impedance analysis (Tanita MC-708 MA P) and estimated to nearest 0.1%. Blood pressure was measured in the sitting position after rest using an automatic blood pressure monitor (Omron HPB-1300, Lidingö, Sweden). Systolic blood pressure (SBP) and diastolic blood pressure (DBP) were estimated by averaging three measurements, taken 1 minute apart. Serum triglycerides (TG) and high-density lipoprotein cholesterol (HDL) were analyzed from blood samples collected during the clinical examination. From the serum measurements, the ratio between TG/HDL was calculated and used as an estimate of whole-body insulin sensitivity.^34^

At the 26-year follow-up, a similar clinical examination was conducted and BMI, body fat %, and blood pressure were reassessed. Blood samples were analyzed for glycosylated hemoglobin (HbA1c) by capillary electrophoresis, to assess long-term glycemic status.

### Assessment of Covariates

Potential confounders for the association between snus use and cardiometabolic markers were selected using directed acyclic graphs, see Figure S2. Education level, occupation, sedentary level, other tobacco use, and secondhand smoke (SHS) exposure were obtained from questionnaire data at 24 years. Physical activity was also assessed at 24 years but was ultimately excluded due to a large portion of missing data (17%). From the baseline questionnaire in BAMSE (conducted at approximately 2 months of age), parental smoking, including maternal smoking during pregnancy, was assessed. Area-level income was assessed at the latest known address of participants at the 24-year follow-up using data from Statistics Sweden on the smallest administrative area units in Sweden, with an average population of 1000–2000 per unit.^35^ Diet was assessed in a separate questionnaire of the 24-year follow-up answered by 1244 participants, from which daily intake of alcohol (g) and total energy (kcal) were calculated. Former tobacco use was defined as either former smoking or former snus use. See Supplementary Materials for more detailed variable definitions.

### Statistical Analysis

Descriptive statistics of potential confounders, snus use, and cardiometabolic markers were compared between women and men, using a two-tailed *t*-test, Wilcoxon rank sum test, or chi-squared test, as appropriate. As both snus use and cardiometabolic markers were strongly associated with sex, all analyses were stratified and performed separately for women and men.

The associations between snus use at around 24 years and cardiometabolic health markers were assessed by linear regression separately at the 24-year follow-up (including BMI, WC, body fat %, TG/HDL ratio, SBP, and DBP) and at the 26-year follow-up (including BMI, body fat %, HBA1c, SBP, and DBP).

To examine the associations with snus and other tobacco products, we categorized exposures as (1) no tobacco use (reference group), (2) exclusive snus use, (3) snus use in combination with other tobacco products (mixed snus), and (4) other tobacco use (including all users of cigarettes, e-cigarettes, and/or waterpipe). To further explore our primary aim of the independent relationship between snus use and cardiometabolic markers, we excluded all participants who used cigarettes, e-cigarettes, and/or waterpipe (*n* = 532 excluded from the 24-year follow-up and *n* = 224 from the 26-year follow-up). Exclusive snus use was then modeled in three separate ways as (1) no tobacco use versus any snus use (occasional and daily snus use combined); (2) categorized into no tobacco use, occasional or daily snus use; and (3) and as no tobacco use, use of <4 or ≥4 cans/week. The cutoff points were in accordance with previous literature.^8,9^ All models were adjusted for education level (university level or below), occupation (student, working or other), median area-level income (in quartiles), sedentary level (>10, 7–9, or 0–6 hours), SHS in utero or infancy (yes or no), and former tobacco use (yes or no). In the 24-year follow-up, sensitivity analyses were performed among participants with dietary data additionally adjusting for alcohol intake (g/d) and total energy intake (kcal/d). To ensure that the observed results were not a consequence of the smaller sample size, analyses were performed both with and without adjusting for dietary factors in this group. Lastly, to investigate the role of nicotine in the relation between snus and cardiometabolic health, cotinine was treated as an exposure variable in a set of linear regression models in the 24-year follow-up. Cotinine levels were log-transformed due to a skewed distribution. Current users of cigarettes, e-cigarettes, and/or waterpipe were excluded from the cotinine analysis, resulting in a sample of 318 women and 345 men.

All statistics were performed in STATA (version 16; Stata Corp, College Station, TX). Participants with missing data were excluded from the analyses.

## Results

### Description of Study Population

The study populations were generally representative of the original BAMSE cohort with regards to background characteristics (see Table S1); however, the proportion of men was lower at the 24-year (44.3%) and 26-year follow-ups (37.9%) compared to the original cohort (50.5%). The study population is described in Table 1. The mean age was 22.6 (range 20–25) years at the 24-year clinical examination, and 25.8 (range 23–27) years at the 26-year follow-up. Men had significantly higher mean BMI, WC, TG/HDL ratio, and blood pressure than women, while women had higher body fat %. HbA1c levels were similar among men and women. Snus use at the 24-year follow-up is described in detail in Table S2. Snus use was more common among men (21.9% vs. 6.4% of women, *p* < .001) and most snus-using men were daily users (16.8 % daily vs. 5.1% occasional), while the distribution of daily versus occasional users among women was more even (3.1% daily vs. 3.3 % occasional users). Among men, 14.6% were exclusive snus users, 7.3% used snus mixed with other tobacco products (80.8% of whom smoked cigarettes), and 14.6% used other types of tobacco (cigarettes, e-cigarettes, and/or waterpipe). Among women, 4.2% used snus exclusively, 2.2% were mixed snus users (of whom 100% were cigarette smokers), and 22.7% used other types of tobacco. Men started to use snus at an earlier age than women (median 18 years in men vs. 21 years in women, *p* < .001) and consumed more cans per week (median 3 cans/week for men vs. 1 can/week for women, *p* < .001).

**Table 1.** Description of Study Population, Snus Exposure, and Cardiometabolic Markers

	Women (*n* = 1257)	Men (*n* = 999)	
	*n* (%)		*p*-Value, chi^b^
University level education at 24 y	526 (41.9)	331 (33.3)	<.001
Occupation at 24 y			.002
Student	697 (55.5)	502 (50.3)	
Working	482 (38.4)	399 (39.9)	
Other	78 (6.2)	98 (9.8)	
Area-level income^a^			.082
Q1 (0–285 531 SEK)	315 (25.1)	217 (21.7)	
Q2 (285 785–349 139 SEK)	320 (25.5)	235 (23.5)	
Q3 (349 351–419 263 SEK)	313 (24.9)	272 (27.2)	
Q4 (419 970–716 119 SEK)	307 (24.5)	275 (27.5)	
Sedentary level at 24 y			<.001
≥10 h	305 (24.5)	336 (34.0)	
7–9 h	414 (33.3)	306 (31.0)	
0–6 h	525 (42.2)	346 (35.0)	
SHS in utero or during infancy	315 (25.1)	224 (22.4)	.145
Former tobacco use at 24 y	170 (13.5)	178 (17.8)	.005
Cigarette smoking at 24 y	284 (22.6)	173 (17.3)	.002
E-cigarette use at 24 y	31 (2.5)	54 (5.4)	<.001
Waterpipe use at 24 y	25 (2.0)	24 (2.4)	.495
No tobacco use at 24 y	891 (70.9)	634 (63.5)	<.001

Snus use at the 24-year follow-up			
	*n* (%)		*p*-Value, *t*-test
Snus use, exclusive	53 (4.2)	146 (14.6)	<.001
Snus use, mixed^b^	28 (2.2)	73 (7.3)	<.001
Other tobacco use	285 (22.7)	146 (14.6)	<.001
	Median (IQR)		*p*-Value, Wilcoxon rank sum test
Age of regular snus use debut (y)	21 (19–22)	18 (16–20)	<.001
Number of snus cans/wk	1 (0.25–2)	3 (1.25–4)	<.001

Cardiometabolic markers at the 24-year follow-up			
	Women (*n* = 1257)	Men (*n* = 999)	
	Mean (SD)		*p*-Value, *t*-test
Body mass index (kg/m^2^)	22.8 (3.9)	23.5 (3.8)	<.001
Waist circumference (cm)	75.3 (8.9)	84.3 (9.7)	<.001
Body fat %	26.6 (6.3)	16.9 (6.2)	<.001
TG/HDL	0.6 (0.5)	0.9 (0.7)	<.001
Systolic blood pressure (mmHg)	116.6 (9.6)	129.3 (10.8)	<.001
Diastolic blood pressure (mmHg)	74.3 (8.0)	76.8 (8.2)	<.001

Cardiometabolic markers at the 26-year follow-up			
	Women (*n* = 628)	Men (*n* = 383)	
	Mean (SD)		*p*-Value, *t*-test
Body mass index (kg/m^2^)	23.1 (4.4)	24.0 (3.7)	<.001
Body fat %	26.8 (6.6)	18.2 (6.0)	<.001
HbA1c (mmol/mol)	31.4 (2.6)	31.7 (2.6)	.150
Systolic blood pressure (mmHg)	120.9 (9.5)	133.9 (10.8)	<.001
Diastolic blood pressure (mmHg)	79.0 (7.7)	81.4 (8.5)	<.001

Abbreviations: IQR = interquartile range; Q = quartile; SD = standard deviation; TG/HDL = triglyceride/high-density lipoprotein cholesterol ratio.

^a^At latest know address, data from Statistics Sweden.

^b^Concurrent use of snus and any of cigarettes, e-cigarettes, or waterpipe.

### Association Between Snus Use and Cardiometabolic Markers at 24 Years

Table 2, part A, shows the adjusted associations between snus and other tobacco use and cardiometabolic markers in the study population from the 24-year follow-up (*n* = 2256). Among women, exclusive snus use was associated with increased BMI (β: 1.23 kg/m^2^, 95% CI = 0.14, 2.32) and nonsignificantly associated with WC (β: 2.22 cm, 95% CI = −0.31, 4.75) as compared to nontobacco users. The estimates for mixed snus were similar for BMI and WC, although with wider confidence intervals. Other tobacco use than snus was associated with increased BMI (β: 0.82 kg/m^2^, 95% CI = 0.29, 1.35), WC (β: 1.58 cm, 95% CI = 0.35, 2.81), and body fat (β: 1.27 %, 95% CI = 0.40, 2.14). Among men, mixed snus use was associated with increased body fat (β: 1.56 %, 95% CI = 0.03, 3.09) and DBP (β: 2.47 mmHg, 95% CI = 0.46, 4.49), while no associations were observed for exclusive snus use around age 24 years. Additional adjustments for alcohol and total energy intake (*n* = 1244) did not influence the results (see Table S3).

**Table 2.** Associations Between Snus Use at 24 Years and Cardiometabolic Markers at the 24-Year Follow-Up (*n* = 2256) and 26-Year Follow-Up (*n* = 1011)

Part A		Cardiometabolic health markers at 24 y								
		BMI (kg/m^2^)	Waist circumference (cm)	Body fat (%)		TG/HDL ratio		Systolic blood pressure (mmHg)		Diastolic blood pressure (mmHg)
	*n*	β (95% CI)	β (95% CI)	β (95% CI)		β (95% CI)		β (95% CI)		β (95% CI)
Women										
No tobacco use	884	0 (Referent)	0 (Referent)	0 (Referent)		0 (Referent)		0 (Referent)		0 (Referent)
Snus use, exclusive	51	**1.23 (0.14, 2.32)**	2.22 (−0.31, 4.75)	0.75 (−1.07, 2.57)		−0.02 (−0.15, 0.11)		0.05 (−2.70, 2.81)		0.19 (−2.10, 2.48)
Snus use, mixed^a^	27	1.30 (−0.16, 2.77)	2.38 (−1.01, 5.77)	1.40 (−1.01, 3.80)		−0.03 (−0.20, 0.15)		2.45 (−1.25, 6.15)		0.79 (−2.27, 3.86)
Other tobacco use^b^	278	**0.82 (0.29, 1.35)**	**1.58 (0.35, 2.81)**	**1.27 (0.40, 2.14)**		0.02 (−0.04, 0.08)		−0.08 (−1.41, 1.25)		-0.64 (−1.75, 0.46)
Men										
No tobacco use	626	0 (Referent)	0 (Referent)	0 (Referent)		0 (Referent)		0 (Referent)		0 (Referent)
Snus use, exclusive	142	0.20 (-0.51, 0.92)	0.12 (-1.71, 1.95)	−0.10 (−1.26, 1.07)		0.06 (−0.07, 0.19)		−0.00 (−2.03, 2.03)		−0.65 (−2.18, 0.88)
Snus use, mixed^a^	71	0.61 (−0.34, 1.55)	1.42 (−0.98, 3.82)	**1.56 (0.03, 3.09)**		0.03 (−0.14, 0.20)		0.90 (−1.78, 3.58)		**2.47 (0.46, 4.49)**
Other tobacco use^b^	146	−0.65 (−1.34, 0.04)	−**1.87 (**−**3.63, -0.12)**	−1.08 (−2.21, 0.05)		0.06 (−0.07, 0.18)		−0.24 (−2.21, 1.72)		−0.12 (−1.60, 1.36)

Part B		Cardiometabolic health markers at 26 years								
		BMI (kg/m^2^)		Body fat (%)	HbA1c (mmol/mol)		Systolic blood pressure (mmHg)		Diastolic blood pressure (mmHg)	
	n	β (95% CI)		β (95% CI)	β (95% CI)		β (95% CI)		β (95% CI)	
Women										
No tobacco use	455	0 (Referent)		0 (Referent)	0 (Referent)		0 (Referent)		0 (Referent)	
Snus use, exclusive	29	1.11 (−0.51, 2.73)		−0.50 (−2.98, 1.98)	0.09 (−0.91, 1.09)		−1.09 (−4.72, 2.54)		−0.03 (−2.99, 2.94)	
Snus use, mixed^a^	11	**3.57 (1.01, 6.13)**		3.78 (−0.14, 7.70)	−0.02 (−1.58, 1.53)		4.92 (−0.82, 10.67)		−0.18 (−4.87, 4.52)	
Other tobacco use^b^	129	0.62 (−0.25, 1.48)		0.89 (−0.44, 2.22)	−0.27 (−0.80, 0.26)		0.20 (−1.75, 2.15)		−0.32 (−1.91, 1.27)	
Men										
No tobacco use	241	0 (Referent)		0 (Referent)	0 (Referent)		0 (Referent)		0 (Referent)	
Snus use, exclusive	56	0.93 (−0.17, 2.03)		0.22 (−1.58, 2.01)	−0.10 (−0.88, 0.68)		0.64 (−2.65, 3.94)		1.08 (−1.54, 3.69)	
Snus use, mixed^a^	31	0.64 (−0.76, 2.03)		0.93 (−1.34, 3.20)	0.16 (−0.82, 1.15)		1.72 (−2.46, 5.90)		1.09 (−2.23, 4.41)	
Other tobacco use^b^	52	−0.53 (−1.65, 0.59)		−0.57 (−2.40, 1.26)	−0.39 (−1.18, 0.41)		−0.70 (−4.07, 2.66)		−0.26 (−2.93, 2.41)	

Abbreviations: BMI = body mass index; CI = confidence interval; SHS = secondhand smoke; TG/HDL = triglyceride/high-density lipoprotein cholesterol ratio. Results from linear regression models adjusted for education level, occupation, sedentary level, area-level income, SHS in utero or infancy and former tobacco use. Statistically significant results (*p* > 0.05) are marked in bold font.

^a^Snus use with concurrent use of other tobacco products.

^b^Cigarettes, e-cigarettes, or waterpipe.

**Figure 1.**
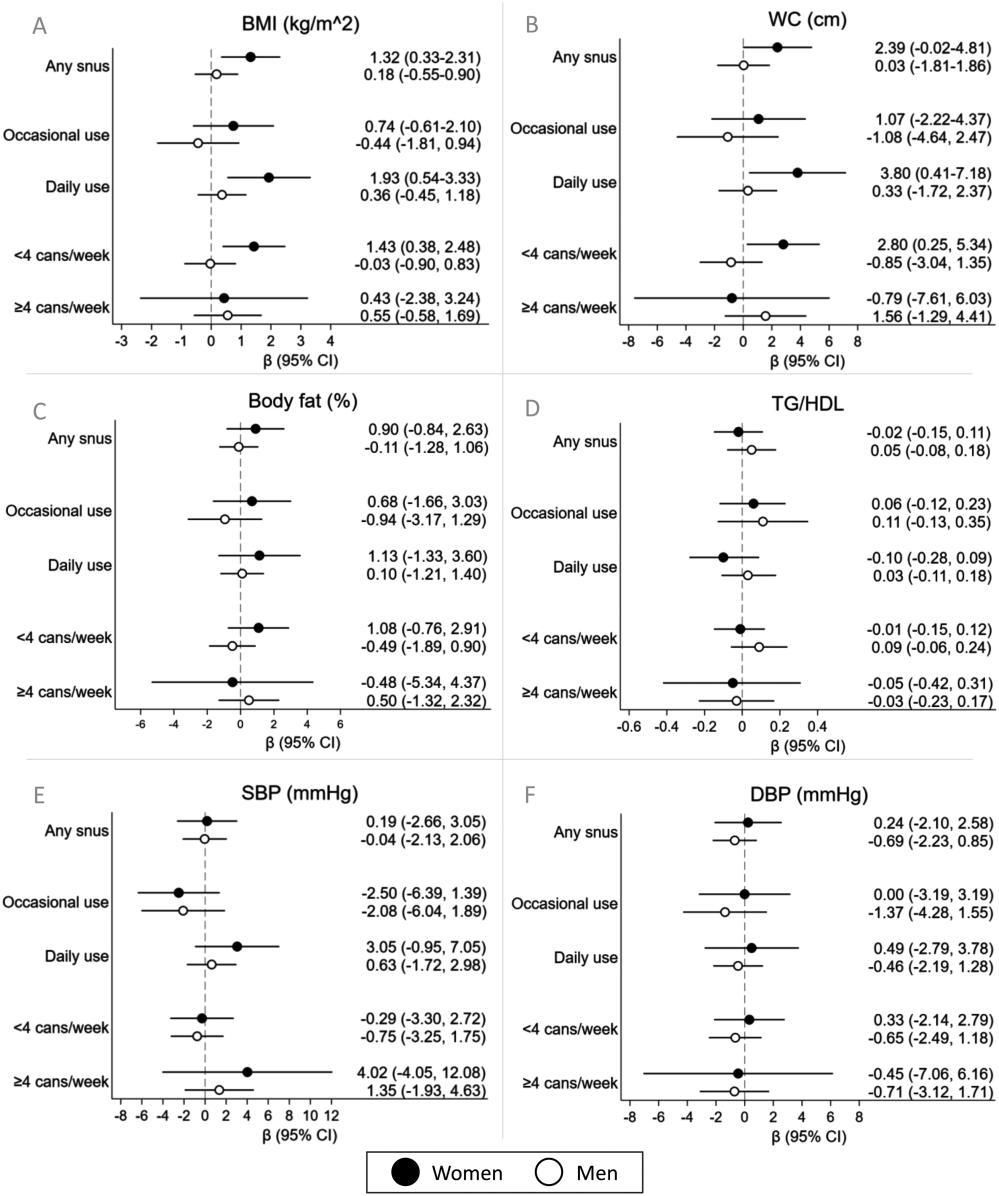
Associations between exclusive snus use at around 24 years and cardiometabolic markers at around 24 years for women and men as compared to nontobacco users for any, occasional, daily, <4 and ≥4 cans/week snus use (*n* = 1724). Results from linear regression models adjusted for education level, occupation, sedentary level, area-level income, SHS in utero or during infancy and former tobacco use. BMI = body mass index; DBP = diastolic blood pressure; TG/HDL = triglyceride/high-density lipoprotein cholesterol ratio; SBP = systolic blood pressure; WC = waist circumference.

Among exclusive snus users (Figure 1), daily use among women was associated with increased BMI (β: 1.93 kg/m^2^, 95% CI = 0.54, 3.33) and WC (β: 3.80 cm, 95% CI = 0.41, 7.18). A tendency toward increased SBP was observed among daily users (β: 3.05 mmHg, 95% CI = −0.95, 7.05). There were no associations observed for occasional snus use or use of ≥4 cans/week, while using <4 cans/week was associated with increased BMI (β: 1.43 kg/m^2^, 95% CI = 0.38, 2.48) and WC (β: 2.80 cm, 95% CI = 0.25, 5.34). For men, no associations were observed between different patterns of snus use and cardiometabolic markers at 24 years. The results for exclusive snus use were also unaffected by additional adjustments for alcohol and total energy intake (see Table S4).

### Association Between Urinary Cotinine and Cardiometabolic Markers at 24 Years

Median urinary cotinine levels among exclusive snus users were 1566 ng/mL among women and 2959 ng/mL among men. Among nontobacco users, the medians were 1.2 and 1.9 ng/mL among women and men, respectively. The association between urinary cotinine levels and cardiometabolic markers at age 24 years is shown in Table S5. Among women, a 1.0-unit increase in log(ng/mL) cotinine was nonsignificantly associated with increased BMI (β: 0.16 kg/m^2^, 95% CI = −0.02, 0.33) and WC (β: 0.36 cm, 95% CI = −0.05, 0.78). No associations were observed for cardiometabolic markers among men, or for body fat %, TG/HDL ratio, or blood pressure among women.

### Association Between Snus Use and Cardiometabolic Markers at 26 Years

Adjusted associations between snus and other tobacco use and cardiometabolic markers at the 26-year follow-up (*n* = 1011) are shown in Table 2, part B. Among women, mixed snus use at 24 years was associated with increased BMI (β: 3.57 kg/m^2^, 95% CI = 1.01, 6.13) and nonsignificantly associated with body fat (β: 3.78 %, 95% CI = −0.14, 7.70) and SBP (β: 4.92 mmHg, 95% CI = −0.82, 10.67), while no associations were observed for exclusive snus use. Among men, exclusive snus users had nonsignificantly increased BMI (β: 0.90 kg/m^2^, 95% CI = −0.17, 2.03), but no other associations were observed between snus or other tobacco use and cardiometabolic markers at 26 years.

**Figure 2.**
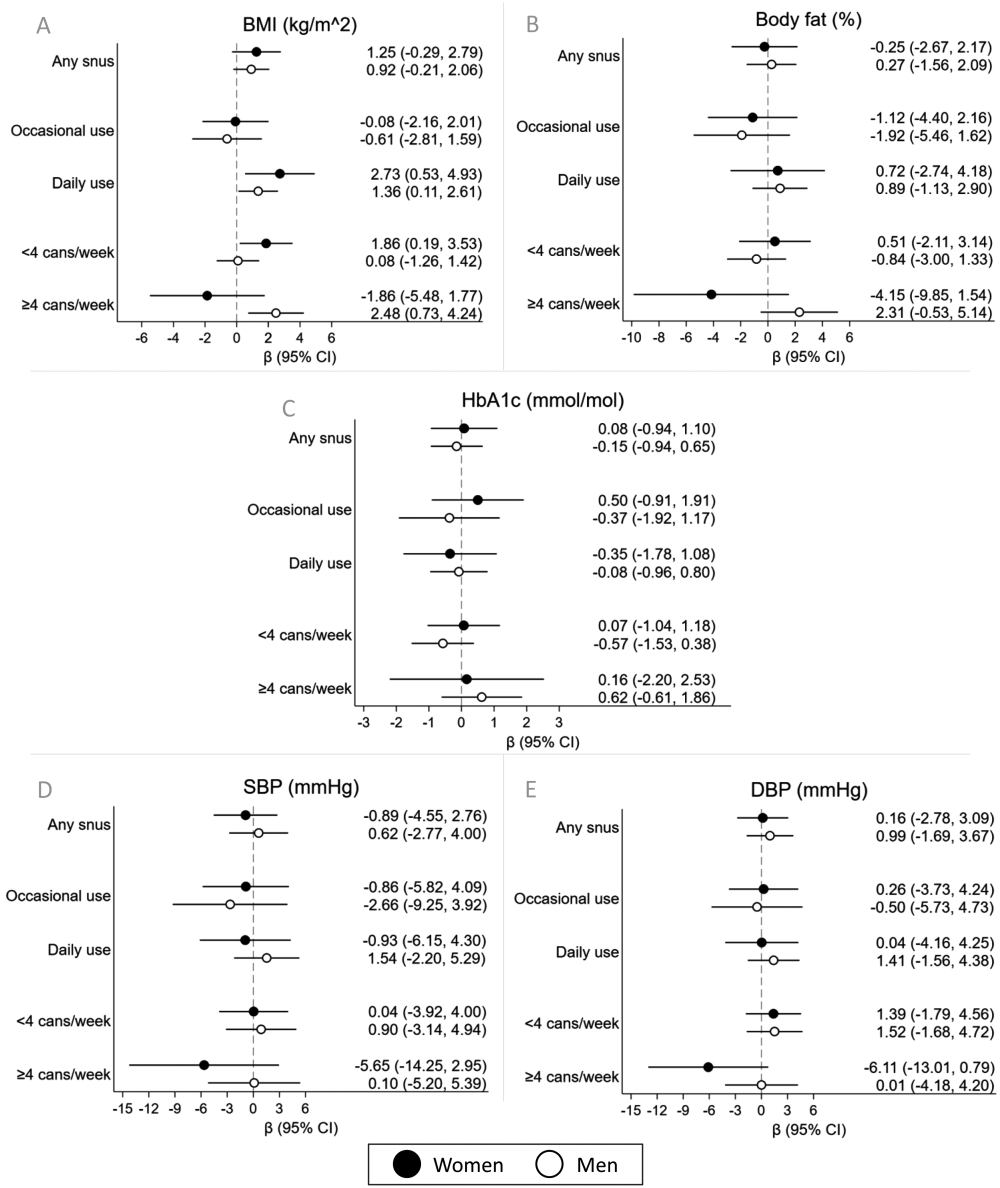
Associations between exclusive snus use at around 24 years and cardiometabolic markers at around 26 years for women and men as compared to nontobacco users, for any, occasional, daily, <4 and ≥4 cans/week snus use (*n* = 787). Results from linear regression models adjusted for education level, occupation, sedentary level, area-level income, SHS in utero or during infancy and former tobacco use. BMI = body mass index; DBP = diastolic blood pressure; HbA1c: glycosylated hemoglobin; TG/HDL = triglyceride/high- density lipoprotein cholesterol ratio; SBP = systolic blood pressure; WC = waist circumference.

Among exclusive snus users (Figure 2), daily snus use among women was associated with increased BMI (β: 2.73 kg/m^2^, 95% CI = 0.53, 4.93), as was using <4 cans/week (β: 1.86 kg/m^2^, 95% CI = 0.19, 3.53). Users of ≥4 cans/week showed tendencies toward lower body fat % and blood pressure at 26 years, although this group consisted of only five women. Among men, increased BMI was observed for daily use (β: 1.36 kg/m^2^, 95% CI = 0.11, 2.61) and use of ≥4 cans/week (β: 2.48 kg/m^2^, 95% CI = 0.73, 4.24). Users of ≥4 cans/week also showed a tendency toward increased body fat (β: 2.31 %, 95% CI = −0.53, 5.14). At 26 years, no significant associations with HbA1c or blood pressure were observed.

## Discussion

In this study of young adults from a prospective Swedish birth cohort, we found associations between exclusive snus use and increased measures of adiposity. In cross-sectional analyses at around 24 years, we observed associations with increased BMI and WC among women. At a later follow-up at around 26 years, snus use was related to increased BMI among both women and men, and heavy-using men tended to have increased body fat %. The observed associations were driven by daily or heavy snus use. Moreover, associations with BMI and WC among women around age 24 years were also indicated using urinary cotinine levels as an objective biomarker of exposure, although these results were not statistically significant. We did not observe any significant associations between snus use and body fat, glycemic status, or blood pressure among young adults.

Previous studies, mainly performed in populations of middle-aged men, have found mixed results on the association between snus and cardiometabolic health markers. In line with our results, snus has been associated with measures of adiposity in several studies. In a Swedish cross-sectional study of middle-aged men, snus was associated with a greater risk of being overweight^17^ and in a longitudinal study of Swedish never-smoking men, using snus at two consecutive follow-ups 5 years apart was associated with weight gain and incident obesity.^16^ In a large Norwegian cohort, extensive snus use (defined as daily snus use for over 5 years, with a monthly consumption above the study population mean) was associated with increased WC and SBP in a middle-aged population.^22^ In a large prospective cohort of 16 000 men and women, heavy snus use (>4 cans/week) was associated with an increased risk of developing metabolic syndrome as well as well as obesity.^9^ On the other hand, in a study on snus use from adolescence to middle age and the risk of metabolic syndrome on 880 participants at age 43, Byhamre and colleagues found that snus use was not associated with central obesity.^27^ Additionally, an increased risk of abdominal obesity was observed in a cross-sectional study of Swedish men, but the associations were confined to former smokers.^36^

We did not find associations between snus and blood pressure. Similarly, snus use was not associated with increased blood pressure in two Swedish studies on metabolic syndrome.^9,27^ On the other hand, snus was associated with hypertension in a large cohort of never-smoking men^21^ as well as with increased blood pressure in a cohort of healthy fire fighters^37^ and among heavy snus users in a Norwegian population-based cohort.^22^

Although snus use, and primarily heavy snus use, has repeatedly been associated with diabetes,^5–7^ snus use has not been observed to be correlated to impaired glucose tolerance or dysglycemia.^9,22,26,27^ This is in line with our results of no association with glycemic status.

It is generally difficult to fully distinguish between the effect of snus and other tobacco products due to the large overlap of usage, also observed in our cohort.^38^ This is an important issue, as cigarette smoking is a strong risk factor for cardiometabolic disease.^1^ In our results, the associations with cardiometabolic health markers largely overlapped for exclusive and mixed use, while some associations, including increased body fat and DBP among men at 24 years, were only observed among multiple tobacco product users, possibly explained by cigarette smoking. In a study on latent autoimmune diabetes in adults and type 2 diabetes in two adult cohorts, both snus use and cigarette smoking were independently associated with increased disease risks.^7^ However, the increased risk estimates were higher for dual users of snus and cigarettes than for snus or cigarette use only. The inconsistencies in the current literature may be due to the lack of distinction between multiple tobacco product users, which is an exposure group that may deserve special attention as well.

When compared to cigarette smoking, snus has been observed to be more strongly associated with cardiometabolic risk factors in several studies. Hansson et al. found that both cigarette smokers and snus users had an increased risk of weight gain over a 5-year period, but only snus users had an increased risk of obesity.^16^ Furthermore, smoking was not associated with metabolic syndrome in a large Swedish cohort, but an increased risk was found for users of >4 boxes of snus/week.^9^ Additionally, current smokers had lower blood pressure and WC compared to snus users in a Norwegian cohort.^22^ In one of few studies on cigarettes and cardiometabolic health among young adults, the authors found similar results to the current study in a Chilean cohort (mean age 22 years). Cigarette smoking was associated with increased BMI and WC at a comparable level to snus use in our cohort, but not with glycemic status or blood pressure.^39^

The mechanisms behind the potential influence of snus on cardiometabolic disease are unclear. Tobacco has a complex relation with weight gain, as cigarette smoking generally decreases weight, and smoking cessation has been observed to lead to weight gain.^40^ The main addictive constituent of both cigarettes and snus, nicotine, contributes to increased energy expenditure in fat cells, but also accumulation of visceral fat.^40^ However, in vitro results indicate that other components in cigarettes and snus contribute to the risk of cardiovascular disease to a higher extent than nicotine alone.^41^ Furthermore, the difference in our results between men and women have previously been difficult to study since few women have used snus in the past. However, experimental studies have observed that snus use significantly increased blood pressure and heart rate among women but not men in a group of young adults.^42^ Women have a higher risk of cardiovascular disease per smoked cigarettes, which may be partly explained by different hormonal effects of nicotine.^43,44^ Moreover, snus and other forms of smokeless tobacco have traditionally been popular among physically fit men,^45^ which may lead to negative confounding and an underestimation of the associations between snus use and cardiometabolic markers.

The current study has several strengths, including being conducted within a well-characterized cohort with a prospective study design and a relatively large study population. Moreover, assessment of height, weight, bioimpedance, and blood pressure was conducted by trained nurses using standardized methods. Furthermore, given the young age of the study population, the occurrence of comorbidities that may affect the investigated health markers can be presumed to be very low. Additionally, the self-reported exposure assessment was supplemented by an objective biomarker for nicotine exposure and has previously been validated against urinary cotinine, finding high validity of self-reported tobacco use, especially among snus users.^33^

Some limitations need to be mentioned as well. The cross-sectional design of the analyses of snus and cardiometabolic outcomes in the 24-year follow-up is a drawback of the study. However, the associations with BMI were confirmed using height and weight collected in the 26-year follow-up. Furthermore, the statistical analysis includes multiple comparisons of outcome markers on the same study sample, which increases the risk of false positive findings. This needs to be taken into consideration when interpreting the study results. Additionally, associations between snus use and cardiometabolic health may be difficult to investigate as several lifestyle factors are likely to correlate with both snus and adverse cardiometabolic health, such as low socioeconomic status and alcohol intake.^46^ Although we adjusted for these and other factors, it is difficult to fully capture possible confounding effects. Moreover, as self-reported tobacco use in the cohort has high specificity but somewhat lower sensitivity,^33^ we expect some minor misclassification in the exposure assessment, which would bias the observed results toward the null. Lastly, although loss-to-follow-up is a potential limitation in all cohort studies, the response rate remained high in our cohort until adulthood and the study populations at 24 and 26 years were comparable to the original BAMSE cohort. As the study population and subpopulation were representative of the full cohort both in terms of snus use and other important characteristics, our observed estimates are unlikely meaningfully affected by selection bias, although there is reduced statistical precision due to the smaller sample size.

The use of snus in the cohort was similar to Swedish national figures for the relevant age groups at the same time period.^28^ Since the 24-year follow-up (2016–2019), snus use has increased among young adults in Sweden, on a population level as well as within the BAMSE cohort.^28,31^ Among women in the cohort, total snus use increased from 6% to 19%,^31^ which may be related to the introduction of a new tobacco product, nicotine pouches or “all white snus,” which has been heavily marketed toward young adults and young women in particular.^47^ However, since we did not specifically ask about this new tobacco product, we could not distinguish between regular and all white snus in our study. Similar trends of increased snus use have been observed in other Nordic countries, and sales of snus in the United States have also increased in the past years.^48,49^ These trends are cause for concern given the possible effect on cardiometabolic disease taken together with the high burden of cardiometabolic outcomes on a societal level.^50^

## Conclusions

In conclusion, we found associations between snus use and increased measures of adiposity among Swedish young adults, in particular, increased BMI and WC among women and BMI among heavy-using (≥4 cans/week) men. We did not find associations between snus use and other markers of cardiometabolic health. These findings provide some insight into the previously largely unstudied effect of snus on health among women. Although the findings from our cross-sectional analyses must be interpreted with caution, more research is warranted to further understand the effect of snus on cardiometabolic health, in particular, given the recent increase in the use of snus among young women.

## Supplementary material

Supplementary material is available at *Nicotine and Tobacco Research* online.

ntae267_suppl_Supplementary_Figures_S1-S2_Tables_S1-S5

## Data Availability

Data not publicly available.
