# Supplementary material for: Snus and Cardiometabolic Health Markers Among Swedish Young Adults
Source: Nicotine Tob Res. 2024 Nov 15;27(6):1098–108. doi: 10.1093/ntr/ntae267 (PMC12095809; doi:10.1093/ntr/ntae267)
Supplement: ntae267_suppl_Supplementary_Figures_S1-S2_Tables_S1-S5 [file ntae267_suppl_supplementary_figures_s1-s2_tables_s1-s5.docx]

Snus and cardiometabolic health markers among Swedish young adults

# Supplementary information

List of content

Page 2 Variable definitions

Page 3 Figure S1. Flow chart of study sample selection

Page 4 Figure S2. Directed acyclic diagram of relationship between snus, cardiometabolic health and possible confounders.

Page 5 Table S1. Descriptive statistics of BAMSE cohort, study population and subpopulation

Page 6 Table S2. Detailed description of snus use at the 24-year follow-up.

Page 7 Table S3. Sensitivity analysis - association between snus use and cardiometabolic markers at 24 years, restricted to participants with dietary data.

Page 8 Table S4. Sensitivity analysis - association between snus use and cardiometabolic markers at 24 years, restricted to participants with dietary data and excluding other tobacco use.

Page 9 Table 5. Association between urinary cotinine levels and cardiometabolic markers at 24 years.

# Variable definitions:

**Education level:** Participants highest completed education level at the time of the 24-year follow-up questionnaire. Categorized as 1) elementary school or high school and 2) university or higher.

**Occupation:** Based on questionnaire data from 24-year follow-up. Categorized as 1) student, 2) working or 3) other.

**Area level income:** Median area level income during 2014 at latest known address at 24 years. Data obtained from Statistics Sweden.

**Sedentary level:** Self-reported sitting time per day, excluding sleep, at 24 years in categories: 1) 10 or more hours, 2) 7-9 hours or 3) 6 or fewer hours.

**Secondhand smoke (SHS) exposure in utero or during infancy:** Mother smoking at least 1 cigarette/day at any point during pregnancy and/or any parent smoking at baseline of the BAMSE cohort. Based on self-reported questionnaire data from parents.

**Former tobacco use:** Either self-reported previous use of cigarettes or snus at age 24, or self-reported daily tobacco use or smoking more than 7 cigarettes/week at the BAMSE 16 follow-up.

Bamse 24 cohort
n=3064

Did not attend the clinical examination
n=794

Participants with full tobacco data
n=2264

**24-year follow-up population
n=2256**

Participants with missing tobacco data
n=6

Attendees of 24-year follow-up clinical examination
n=2270

Excluding pregnant participants
n=8

Did not attend COVID-19 follow-up clinical examination
n=1232

Attendees of BAMSE C19 clinical examination n=1024

Participants with missing tobacco data or pregnant
n=13

**26-year follow-up population
n=1011**

Figure S1. Flow chart of study sample selection, Study population and subpopulation

Alt text Figure S1: Figure describing the selection process of the study population and subpopulation from the original BAMSE cohort.


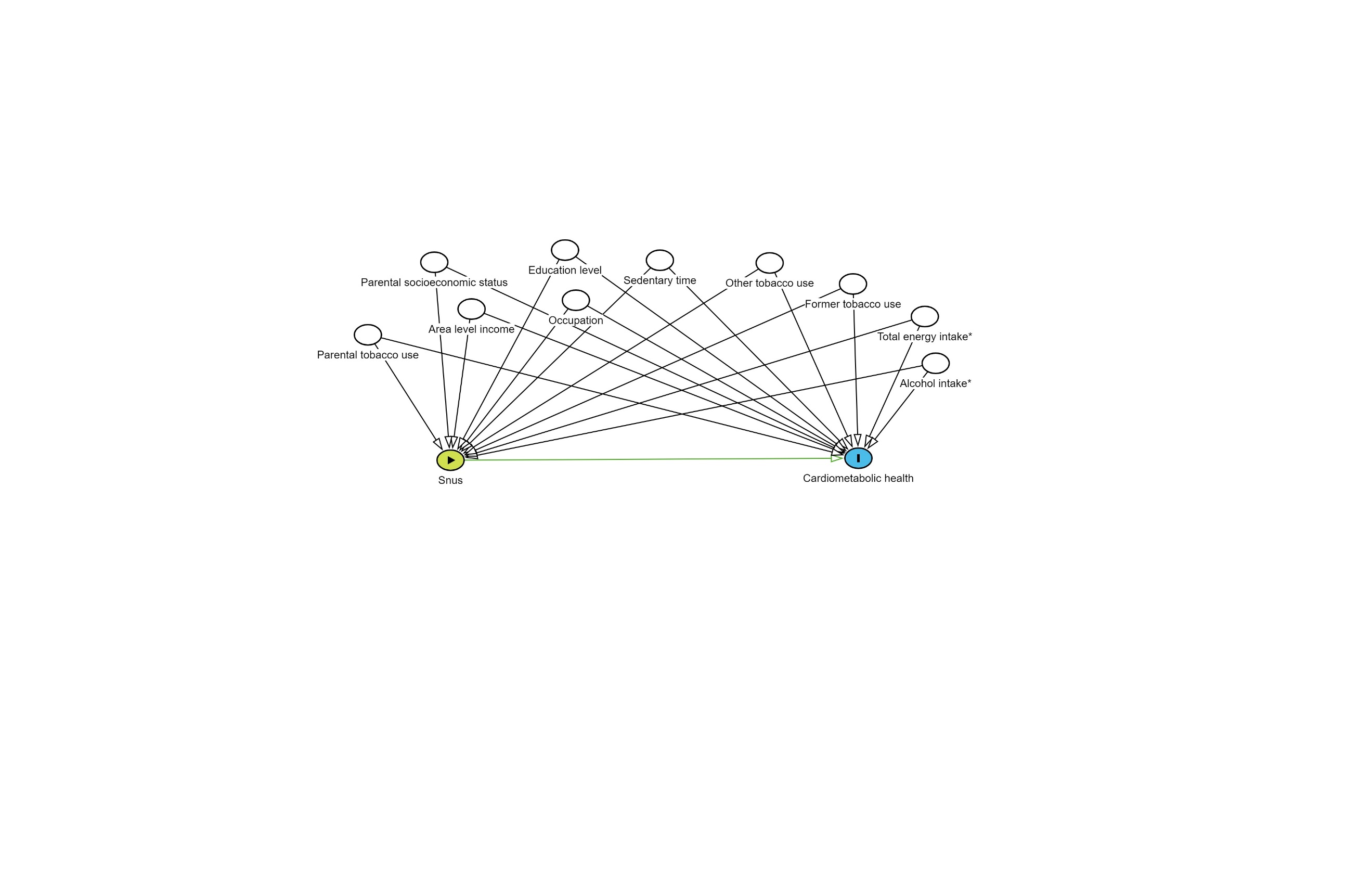


Figure S2. DAG of relationship between snus, cardiometabolic health and possible confounders in the BAMSE cohort. *Confounders adjusted for in sensitivity analysis. Created in DAGitty v3.1.

Alt text Figure S2: Figure describing the schematic relationship between exposure to snus and cardiometabolic health outcomes in relation to the following confounding factors: Parental tobacco use, parental socioeconomic status, area level income, education level, occupation, sedentary time, other tobacco use, former tobacco use, total energy intake and alcohol intake.

Table S1. Descriptive statistics of the BAMSE cohort, study population and subpopulation

Percentages may not add up, due to missing values

|  | **Full BAMSE cohort**  **n=4089** | **24-year follow-up**  **n=2256** | **26-year follow-up**  **n=1011** |
| --- | --- | --- | --- |
|  | **n (%)** | **n (%)** | **n (%)** |
| Male sex | 2065 (50.5) | 999 (44.3) | 383 (37.9) |
| University level education at age 24 | 1 096 (36.0) | 857 (38.1) | 397 (39.4) |
| *Occupation at age 24:* |  |  |  |
| Student | 1 564 (51.2) | 1 199 (53.2) | 578 (57.2) |
| Working | 1 250 (40.9) | 881 (39.1) | 368 (36.4) |
| Other | 240 (7.9) | 176 (7.8) | 65 (6.4) |
| *Area level income at latest address in 2014:* |  |  |  |
| Q1 (0 - 285 531 SEK) | 1 022 (25.0) | 532 (23.6) | 246 (24.3) |
| Q2 (285 785 - 349 139 SEK) | 1 019 (25.0) | 555 (24.6) | 242 (23.9) |
| Q3 (349 351 - 419 263 SEK) | 1 021 (25.0) | 585 (26.0) | 256 (25.3) |
| Q4 (419 970 - 716 119 SEK) | 1 019 (25.0) | 582 (25.8) | 267 (26.4) |
| *Sedentary level at age 24:* |  |  |  |
| $\geq$10 hours/day | 827 (28.5) | 641 (28.7) | 287 (28.6) |
| 7-9 hours/day | 921 (31.8) | 720 (32.3) | 330 (32.8) |
| 0-6 hours/day | 1 151 (39.7) | 871 (39.0) | 388 (38.6) |
| Smoke exposure in utero or during infancy | 1 012 (24.8) | 539 (23.9) | 224 (22.2) |
| Former tobacco use (at age 24) | 465 (15.2) | 348 (15.4) | 149 (14.7) |
| Snus use (at age 24) | 478 (15.6) | 300 (13.3) | 129 (12.8) |
| Cigarette smoking (at age 24) | 633 (20.7) | 457 (20.3) | 191 (18.9) |
| E-cigarette use (at age 24) | 120 (3.9) | 86 (3.8) | 32 (3.2) |
| Waterpipe use (at age 24) | 62 (2.1) | 49 (2.2) | 21 (2.1) |
| Mixed tobacco use (at age 24) | 218 (20.8) | 145 (19.8) | 56 (18.0) |
| No tobacco use (at age 24) | 2015 (65.8) | 1525 (67.6) | 700 (69.2) |

Table S2. Detailed description of snus use at the 24-year follow-up in the study populations at the 24-year and 26-year follow-up.

|  | **24-year follow-up** | | |  | **26-year follow-up** | | |
| --- | --- | --- | --- | --- | --- | --- | --- |
|  | **Women**  **(n=1257)** | **Men**  **(n=999)** |  |  | **Women**  **(n=628)** | **Men**  **(n=383)** |  |
|  | **n (%)** | **n (%)** | **p-value (chi^2^)** |  | **n (%)** | **n (%)** | **p-value (chi^2^)** |
| **Snus use, total** | 81 (6.4) | 219 (21.9) | <0.001 |  | 41 (6.5) | 88 (23.0) | <0.001 |
| Occasional use | 42 (3.3) | 51 (5.1) | 0.004 |  | 21 (3.3) | 21 (5.5) | 0.098 |
| Daily use | 39 (3.1) | 168 (16.8) | <0.001 |  | 20 (3.2) | 67 (17.5) | <0.001 |
| <4 cans/week | 70 (5.6) | 137 (13.7) | <0.001 |  | 34 (5.4) | 55 (14.4) | <0.001 |
| ≥4 cans/week | 11 (0.9) | 81 (8.1) | <0.001 |  | 7 (1.1) | 33 (8.6) | <0.001 |
| **Snus use, exclusive** | 53 (4.2) | 146 (14.6) | <0.001 |  | 30 (4.8) | 57 (14.9) | <0.001 |
| Occasional use | 27 (2.1) | 32 (3.2) | 0.119 |  | 16 (2.6) | 12 (3.1) | 0.582 |
| Daily use | 26 (2.1) | 114 (11.4) | <0.001 |  | 14 (2.2) | 45 (11.8) | <0.001 |
| <4 cans/week | 46 (3.7) | 92 (9.2) | <0.001 |  | 25 (4.0) | 37 (9.7) | <0.001 |
| ≥4 cans/week | 7 (0.6) | 54 (5.4) | <0.001 |  | 5 (0.8) | 20 (5.2) | <0.001 |
| **Snus use, mixed** | 28 (2.2) | 73 (7.3) | <0.001 |  | 11 (1.8) | 31 (8.1) | <0.001 |
| Other tobacco use among snus users | |  |  |  |  |  |  |
| Cigarettes | 28 (100) | 59 (80.8) | 0.013 |  | 11 (100) | 24 (77.4) | 0.084 |
| E-cigarettes | 0 (0) | 21 (28.8) | 0.001 |  | 0 (0) | 8 (25.8) | 0.061 |
| Waterpipe | 1 (3.6) | 6 (8.5) | 0.394 |  | 0 (0) | 5 (16.1) | 0.156 |

Table S3. Associations between snus use at 24 years and cardiometabolic markers at the 24-year follow-up, restricted to participant with dietary data. Model 1 controlled for education level, occupation, sedentary level, area level income, SHS in utero or infancy and former tobacco use. Model 2 was additionally adjusted for alcohol intake and total energy intake.

| **Women (n=782)** | | | | | | | | | | | | |
| --- | --- | --- | --- | --- | --- | --- | --- | --- | --- | --- | --- | --- |
|  | **BMI (kg/m^2^)** | | **Waist circumference (cm)** | | **Body fat (%)** | | **TG/HDL ratio** | | **Systolic blood pressure (mmHg)** | | **Diastolic blood pressure (mmHg)** | |
|  | Model 1  β (95% CI) | Model 2  β (95% CI) | Model 1  β (95% CI) | Model 2  β (95% CI) | Model 1  β (95% CI) | Model 2  β (95% CI) | Model 1  β (95% CI) | Model 2  β (95% CI) | Model 1  β (95% CI) | Model 2  β (95% CI) | Model 1  β (95% CI) | Model 2  β (95% CI) |
| No tobacco use n=589 | 0 (Referent) | 0 (Referent) | 0 (Referent) | 0 (Referent) | 0 (Referent) | 0 (Referent) | 0 (Referent) | 0 (Referent) | 0 (Referent) | 0 (Referent) | 0 (Referent) | 0 (Referent) |
| Snus, exclusive n=30 | 2.50 (1.19, 3.80)* | 2.60 (1.29, 3.92)* | 5.77 (2.67, 8.87)* | 5.56 (2.44, 8.69)* | 1.78 (-0.52, 4.07) | 1.75 (-0.58, 4.08) | 0.04 (-0.12, 0.19) | 0.07 (-0.09, 0.22) | 0.45 (-3.10, 3.99) | 0.32 (-3.29, 3.92) | 1.07 (-1.93, 4.08) | 1.01 (-1.42, 3.44) |
| Snus, mixed^a^ n=19 | 0.80 (-0.80, 2.41) | 0.89 (-0.74, 2.53) | 1.56 (-2.27, 5.38) | 1.08 (-2.80, 4.96) | 0.49 (-2.28, 3.27) | 0.40 (-2.44, 3.24) | -0.04 (-0.22, 0.15) | 0.00 (-0.19, 0.19) | 2.12 (-2.25, 6.50) | 1.94 (-2.54, 6.42) | 0.45 (-3.26, 4.16) | 1.00 (-2.47, 4.47) |
| Other tobacco^b^ n=144 | 0.50 (-0.15, 1.16) | 0.61 (-0.06, 1.29) | 0.72 (-0.85, 2.28) | 0.67 (-0.94, 2.27) | 0.81 (-0.33, 1.95) | 0.83 (-0.35, 2.01) | 0.07 (-0.01, 0.14) | 0.09 (0.01, 0.17)* | -0.23 (-2.02, 1.55) | -0.33 (-2.18, 1.52) | -1.08 (-2.60, 0.44) | 0.52 (-2.05, 3.10) |
| **Men (n=449)** | | | | | | | | | | | | |
|  | **BMI (kg/m^2^)** | | **Waist circumference (cm)** | | **Body fat (%)** | | **TG/HDL ratio** | | **Systolic blood pressure (mmHg)** | | **Diastolic blood pressure (mmHg)** | |
|  | Model 1  β (95% CI) | Model 2  β (95% CI) | Model 1  β (95% CI) | Model 2  β (95% CI) | Model 1  β (95% CI) | Model 2  β (95% CI) | Model 1  β (95% CI) | Model 2  β (95% CI) | Model 1  β (95% CI) | Model 2  β (95% CI) | Model 1  β (95% CI) | Model 2  β (95% CI) |
| No tobacco use  n=321 | 0 (Referent) | 0 (Referent) | 0 (Referent) | 0 (Referent) | 0 (Referent) | 0 (Referent) | 0 (Referent) | 0 (Referent) | 0 (Referent) | 0 (Referent) | 0 (Referent) | 0 (Referent) |
| Snus, exclusive n=57 | 1.28 (0.31, 2.26)* | 1.42 (0.41, 2.43)* | 2.44 (-0.28, 5.17) | 2.48 (-0.34, 5.31) | 1.47 (-0.21, 3.15) | 1.75 (0.02, 3.47)* | 0.08 (-0.12, 0.27) | 0.11 (-0.09, 0.31) | 3.10 (-0.26, 6.46) | 3.70 (0.23, 7.18)* | 1.26 (-1.80, 4.31) | 1.23 (-1.29, 3.75) |
| Snus, mixed^a^ n=24 | -0.12 (-1.51, 1.28) | 0.13 (-1.31, 1.58) | 0.12 (-3.75, 3.98) | 0.25 (-3.77, 4.26) | 0.11 (-2.28, 2.51) | 0.71 (-1.75, 3.18) | -0.15 (-0.43, 0.12) | -0.12 (-0.41, 0.17) | -0.50 (-5.30, 4.30) | 0.20 (-4.78, 5.17) | 0.77 (-3.03, 4.57) | 1.28 (-2.33, 4.89) |
| Other tobacco^b^ n=47 | -0.36 (-1.39, 0.67) | -0.29 (-1.36, 0.78) | -1.69 (-4.56, 1.17) | -1.77 (-4.74, 1.20) | -0.68 (-2.46, 1.09) | -0.64 (-2.46, 1.18) | 0.00 (-0.20, 0.21) | 0.04 (-0.17, 0.25) | 1.35 (-2.21, 4.91) | 2.14 (-1.54, 5.81) | -0.97 (-2.54, 0.60) | 0.79 (-1.87, 3.46)s |

^a^: Snus use with concurrent use of other tobacco products
^b^: Cigarettes, e-cigarettes or waterpipe
*: p<0.05Table S4. Associations between snus use at 24 years and cardiometabolic markers at the 24-year follow-up, restricted to participant with dietary data and excluding all users or cigarettes, e-cigarettes and waterpipe. Model 1 controlled for education level, occupation, sedentary level, area level income, SHS in utero or infancy and former tobacco use. Model 2 was additionally adjusted for alcohol intake and total energy intake.

| **Women (n=782)** | | | | | | | | | | | | |
| --- | --- | --- | --- | --- | --- | --- | --- | --- | --- | --- | --- | --- |
|  | **BMI (kg/m^2^)** | | **Waist circumference (cm)** | | **Body fat (%)** | | **TG/HDL ratio** | | **Systolic blood pressure (mmHg)** | | **Diastolic blood pressure (mmHg)** | |
|  | Model 1  β (95% CI) | Model 2  β (95% CI) | Model 1  β (95% CI) | Model 2  β (95% CI) | Model 1  β (95% CI) | Model 2  β (95% CI) | Model 1  β (95% CI) | Model 2  β (95% CI) | Model 1  β (95% CI) | Model 2  β (95% CI) | Model 1  β (95% CI) | Model 2  β (95% CI) |
| No tobacco use n=589 | 0 (Referent) | 0 (Referent) | 0 (Referent) | 0 (Referent) | 0 (Referent) | 0 (Referent) | 0 (Referent) | 0 (Referent) | 0 (Referent) | 0 (Referent) | 0 (Referent) | 0 (Referent) |
| Snus, any n=30 | 2.50 (1.25, 3.74)* | 2.73 (1.47, 4.00)* | 5.79 (2.78, 8.81)* | 5.83 (2.76, 8.91)* | 1.75 (-0.47, 3.97) | 1.85 (-0.42, 4.13) | 0.03 (-0.11, 0.17) | 0.06 (-0.09, 0.21) | 0.43 (-3.19, 4.05) | 0.08 (-3.63, 3.80) | 1.04 (-2.01, 4.10) | 1.07 (-2.07, 4.20) |
| Occasional use  n=14 | 1.41 (-0.37, 3.18) | 1.68 (-0.11, 3.47) | 4.09 (-0.21, 8.40) | 4.18 (-0.17, 8.53) | 1.62 (-1.49, 4.73) | 1.75 (-1.41, 4.91) | 0.12 (-0.08, 0.33) | 0.16 (-0.05, 0.36) | -0.76 (-5.93, 4.41) | -1.15 (-6.40, 4.11) | 0.71 (-3.66, 5.08) | 0.72 (-3.72, 5.16) |
| Daily use  n=16 | 3.48 (1.79, 5.17)* | 3.67 (1.97, 5.37)* | 7.32 (3.23, 11.42)* | 7.31 (3.18, 11.43)* | 1.87 (-1.19, 4.93) | 1.95 (-1.15, 5.05) | -0.06 (-0.26, 0.14) | -0.03 (-0.23, 0.16) | 1.51 (-3.41, 6.42) | 1.18 (-3.79, 6.16) | 1.34 (-2.81, 5.50) | 1.37 (-2.83, 5.58) |
| <4 cans/week   n= 28 | 2.57 (1.29, 3.85)* | 2.76 (1.47, 4.06)* | 6.01 (2.91, 9.11)* | 5.99 (2.84, 9.13)* | 1.76 (-0.53, 4.04) | 1.84 (-0.49, 4.17) | 0.04 (-0.10, 0.19) | 0.07 (-0.08, 0.22) | 0.26 (-3.46, 3.98) | -0.05 (-3.85, 3.75) | 0.85 (-2.29, 4.00) | 0.90 (-2.31, 4.11) |
| ≥4 cans/week   n=2 | 1.39 (-3.31, 6.10) | 2.23 (-2.51, 6.96) | 2.48 (-8.92, 13.88) | 3.28 (-8.21, 14.77) | 1.63 (-6.60, 9.86) | 2.03 (-6.30, 10.36) | -0.15 (-0.68, 0.39) | -0.07 (-0.61, 0.46) | 3.09 (-10.60, 16.78) | 2.35 (-11.51, 16.21) | 3.97 (-7.59, 15.53) | 3.87 (-7.83, 15.58) |
| **Men (n=449)** | | | | | | | | | | | | |
|  | **BMI (kg/m^2^)** | | **Waist circumference (cm)** | | **Body fat (%)** | | **TG/HDL ratio** | | **Systolic blood pressure (mmHg)** | | **Diastolic blood pressure (mmHg)** | |
|  | Model 1  β (95% CI) | Model 2  β (95% CI) | Model 1  β (95% CI) | Model 2  β (95% CI) | Model 1  β (95% CI) | Model 2  β (95% CI) | Model 1  β (95% CI) | Model 2  β (95% CI) | Model 1  β (95% CI) | Model 2  β (95% CI) | Model 1  β (95% CI) | Model 2  β (95% CI) |
| No tobacco use  n=321 | 0 (Referent) | 0 (Referent) | 0 (Referent) | 0 (Referent) | 0 (Referent) | 0 (Referent) | 0 (Referent) | 0 (Referent) | 0 (Referent) | 0 (Referent) | 0 (Referent) | 0 (Referent) |
| Snus, any n=57 | 1.34 (0.35, 2.34)* | 1.35 (0.31, 2.38)* | 2.36 (-0.39, 5.11) | 2.21 (-0.65, 5.06) | 1.65 (-0.05, 3.35) | 1.76 (0.01, 3.51)* | 0.07 (-0.13, 0.28) | 0.11 (-0.10, 0.32) | 2.96 (-0.50, 6.41) | 3.74 (0.15, 7.33)* | 0.80 (-1.62, 3.23) | 1.33 (-1.19, 3.86) |
| Occasional use  n=10 | 1.15 (-1.01, 3.31) | 1.11 (-1.09, 3.30) | 2.55 (-3.68, 8.78) | 2.35 (-3.97, 8.67) | 2.16 (-1.51, 5.83) | 2.21 (-1.48, 5.91) | 0.22 (-0.22, 0.65) | 0.27 (-0.17, 0.72) | 1.82 (-5.65, 9.29) | 2.99 (-4.61, 10.58) | 0.73 (-4.51, 5.98) | 1.50 (-3.84, 6.84) |
| Daily use  n=47 | 1.39 (0.30, 2.47)* | 1.40 (0.28, 2.51)* | 2.32 (-0.64, 5.29) | 2.18 (-0.87, 5.23) | 1.54 (-0.31, 3.38) | 1.66 (-0.22, 3.54) | 0.04 (-0.18, 0.26) | 0.08 (-0.15, 0.30) | 3.21 (-0.55, 6.96) | 3.90 (0.04, 7.75)* | 0.82 (-1.82, 3.45) | 1.30 (-1.41, 4.01) |
| <4 cans/week   n=37 | 1.19 (-0.01, 2.38) | 1.18 (-0.08, 2.43) | 1.55 (-1.76, 4.85) | 1.27 (-2.19, 4.74) | 1.42 (-0.61, 3.45) | 1.54 (-0.57, 3.66) | 0.18 (-0.06, 0.42) | 0.25 (-0.01, 0.50) | 2.47 (-1.66, 6.60) | 3.49 (-0.85, 7.83) | 0.90 (-2.00, 3.80) | 1.63 (-1.42, 4.68) |
| ≥4 cans/week   n=20 | 1.63 (0.06, 3.21)* | 1.63 (0.05, 3.20)* | 3.83 (-0.46, 8.13) | 3.74 (-0.57, 8.05) | 2.08 (-0.59, 4.76) | 2.11 (-0.54, 4.76) | -0.13 (-0.45, 0.18) | -0.11 (-0.43, 0.20) | 3.86 (-1.59, 9.30) | 4.16 (-1.30, 9.61) | 0.62 (-3.20, 4.44) | 0.83 (-3.00, 4.67) |

*: p<0.05

Table S5. Association between log-transformed urinary cotinine level at 24 years and cardiometabolic markers at the 24-year follow-up among non-tobacco users and exclusive snus users. The analyses were adjusted for education level, occupation, sedentary level, area level income, SHS in utero or during infancy and former tobacco use. TG/HDL x10: triglyceride/high density lipoprotein cholesterol ratio multiplied by 10.

| **Cotinine (log(ng/ml)) at 24 years**  **β (95% CI)** | | | | |
| --- | --- | --- | --- | --- |
|  | Women (n=318) | | Men (n=345) | |
|  | *Crude* | *Adjusted* | *Crude* | *Adjusted* |
| Body mass index (kg/m^2^) | 0.14 (-0.02, 0.29) | 0.16 (-0.02, 0.33) | -0.01 (-0.11, 0.09) | -0.03 (-0.14, 0.08) |
| Waist circumference (cm) | 0.28 (-0.10, 0.67) | 0.36 (-0.05, 0.78) | -0.03 (-0.30, 0.23) | -0.07 (-0.35, 0.22) |
| Body fat % | 0.07 (-0.19, 0.32) | 0.07 (-0.21, 0.35) | -0.09 (-0.26, 0.09) | -0.10 (-0.29, 0.09) |
| TG/HDL x10 | -0.08 (-0.26, 0.10) | -0.08 (-0.28, 0.12) | 0.02 (-0.16, 0.20) | -0.03 (-0.23, 0.16) |
| Systolic blood pressure (mmHg) | 0.12 (-0.29, 0.52) | 0.21 (-0.24, 0.66) | 0.03 (-0.27, 0.32) | 0.06 (-0.27, 0.39) |
| Diastolic blood pressure (mmHg) | -0.05 (-0.37, 0.27) | 0.09 (-0.26, 0.45) | 0.00 (-0.22, 0.22) | -0.01 (-0.25, 0.24) |
